# Respiratory modulations in the photoplethysmogram (DPOP) as a measure of respiratory effort

**DOI:** 10.1007/s10877-015-9763-y

**Published:** 2015-09-16

**Authors:** Paul S. Addison

**Affiliations:** Respiratory and Monitoring Solutions, Medtronic, Technopole Centre, Edinburgh, EH26 0PJ UK

**Keywords:** Hemodynamic monitoring, Fluid responsiveness, Pulse oximetry, DPOP, PPV

## Abstract

DPOP is a measure of the strength of respiratory modulations present in the pulse oximetry photoplethysmogram (pleth) waveform. It has been proposed as a non-invasive parameter for the prediction of the response to volume expansion in hypovolemic patients. The effect of resistive breathing on the DPOP parameter was studied to determine whether it may have an adjunct use as a measure of respiratory effort. Healthy volunteers were tasked to breathe at fixed respiratory rates over a range of airway resistances generated by a flow resistor inserted within a mouthpiece. Changes in respiratory efforts, effected by the subjects and measured as airway pressures at the mouth, were compared to DPOP values derived from a finger pulse oximeter probe. It was found that the increased effort to breathe manifests itself as an associated increase in DPOP. Further, a relationship between DPOP and percent modulation of the pleth waveform was observed. A version of the DPOP algorithm that corrects for low perfusion was implemented which resulted in an improved relationship between DPOP and PPV. Although a limited cohort of seven volunteers was used, the results suggest that DPOP may be useful as a respiratory effort parameter, given that the fluid level of the patient is maintained at a constant level over the period of analysis.

## Introduction

Respiratory effort is a context specific term defined relative to the clinical setting. A range of physiological measurements may be used in its assessment including maximal inspiratory and expiratory pressures [1]; pleural pressures, esophageal pressures and work of breathing [2]; changes in nasal cannula pressures [3]; changes in blood pressures or pulse transit times [4, 5]; changes in EMG signals [6]; chest band extensions [7]; visual observation of signs of distress and muscle use [8]; changes in coloration [9]; and respiratory sounds [10]. It is now recognised that respiratory components clearly manifest in the photoplethysmogram (pleth) across a range of conditions [11–13]. Analysis of the *frequency* of these components has led to the development of respiratory rate (RR) algorithms for incorporation within pulse oximeter devices [14, 15]. However, the *strength* of these modulations also provides information on the thoracic pressure changes, associated with the effort to breathe. Respiratory effort manifests in the pleth waveform as a variety of distinct modulations which has led to the development of various technologies for its measurement [16–19].

**Fig. 1:**
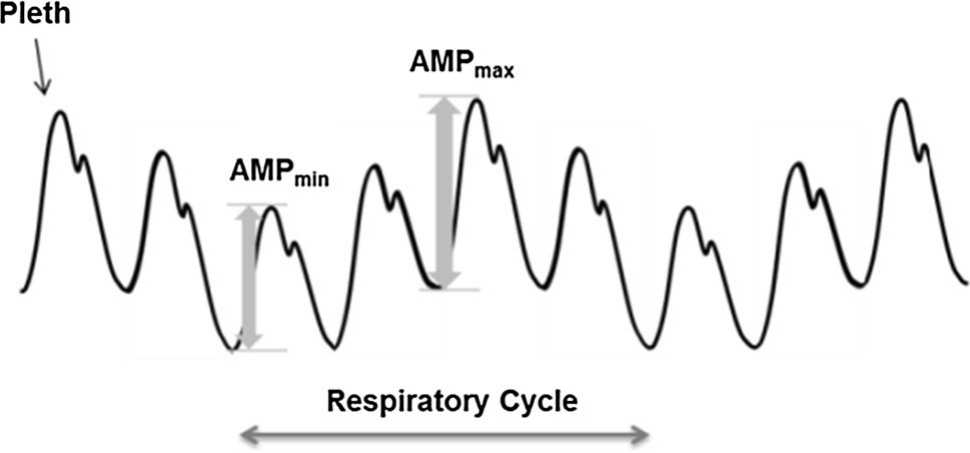
Deriving DPOP from the pleth

This present study explored the potential for using the pulse oximetry photoplethysmographic fluid responsiveness parameter, DPOP, as a measure of respiratory effort. DPOP is a non-invasive measure of the normalized strength of respiratory modulations present in the pleth waveform [20]. It is defined as [21]:

1 $$DPOP = {{\left( {AMP_{max} - AMP_{min} } \right)} \mathord{\left/ {\vphantom {{\left( {AMP_{max} - AMP_{min} } \right)} {\left( {\left( {AMP_{max} + AMP_{min} } \right)/2} \right)}}} \right. \kern-0pt} {\left( {\left( {AMP_{max} + AMP_{min} } \right)/2} \right)}}$$

where *AMP*_*max*_ and *AMP*_*min*_ are the maximum and minimum amplitudes of the cardiac pulse waveforms in the pleth during a respiratory cycle. These are illustrated in Fig. 1, where the cardiac pulse component of the pleth is shown being modulated by respiratory activity. DPOP has been proposed as a measure of the response to fluid administration in mechanically ventilated patients. As such it represents a non-invasive alternative for pulse pressure variation (PPV) [22] with many studies showing favourable correlation between the two parameters [23–29]. DPOP is derived from the change (or delta) in the pulse oximetry photoplethysmogram (POP) waveform.

In this study, data from healthy volunteers undergoing coached breathing in a laboratory setting was employed to study the effect of respiratory effort on DPOP. The DPOP parameter was compared with PPV, derived from the arterial blood pressure waveform which may also be used in practice to determine fluid responsiveness. The effect of low perfusion on the DPOP parameter was also considered where the performance of a modified DPOP parameter, corrected for low perfusion, was studied. This low perfusion correction is fully documented elsewhere [30, 31].

## Methods

### Protocol

With institutional review board approval and written informed consent, seven healthy adult volunteers were enrolled at the Covidien RMS clinical laboratory in Boulder, CO. These volunteers had been pre-screened and completed physical exams. During the trials, the subjects were positioned comfortably in a chair with the pulse oximeter sensor attached (Max-A, Covidien, Boulder, CO). A facemask was attached to each subject consisting of a spirometer containing a number of interchangeable flow resistors [5, 20 and 50 cmH_2_O/l/s linear resistors (Hans Rudolf Inc., Kansas City, MO)]. The blood pressure signal from an intra-arterial line was also recorded. A synchronized acquisition of the pulse oximeter, arterial pressure and airway pressure signals was performed via a custom data acquisition system during the whole procedure and saved to a laptop for later analysis.

The subjects were asked to breathe at a fixed RR of 8 breaths-per-minute (brpm) through the spirometer. This was facilitated by following a metronome displayed on a computer screen next to the subject. Each breathing task was maintained for a 1-min period separated in time by a 2-min recovery period in order to allow the subject to relax and breathe freely in preparation for the next 1-min task.

Each subject was initially asked to breathe through the selected flow resistor at a consistent effort for a 1-min period at what they felt was an appropriate effort for that resistance. This is labelled (M). For the second 1-min effort task, the subject was asked to breathe with more effort through the resistor. This is labelled (H). During the third task the subject was asked to breathe with less effort through the resistor than the initial effort (S), and during the fourth task the subject was asked to breathe in a similar manner to the first attempt (M). The medium (M) efforts allowed the patient to familiarize him/herself with the apparatus and to set a baseline. The maximum (H) and minimum (L) effort breathing values were then extracted for analysis. This provided two 1-min segments of pleth and pressure signals at either extreme of air pressures at the mouthpiece. The equipment also allowed for the option to set the flow resistance only on inhalation or only on exhalation. The regimes undertaken by the seven subjects are summarised in Table 1 where it can be seen that five subjects encountered flow resistance on inhalation and two on exhalation. Each series of tasks was repeated for the three flow resistances—5, 20 and 50 cmH_2_O/l/s. Two subjects had difficulty breathing at the highest flow resistor level of 50 cmH_2_O/l/s and did not complete this task.

**Table 1:** The available subject data sets

Patient ID	Resistor direction	C = 20		C = 5		C = 50	
		Hard	Soft	Hard	Soft	Hard	Soft
RE002	Inhale	Y	Y	Y	Y	N	N
RE003	Exhale	Y	Y	Y	Y	Y	Y
RE005	Inhale	Y	Y	Y	Y	N	N
RE006	Exhale	Y	Y	Y	Y	Y	Y
RE008	Inhale	Y	Y	Y	Y	Y	Y
RE009	Inhale	Y	Y	Y	Y	Y	Y
RE011	Inhale	Y	Y	Y	Y	Y	Y

Flow resistor values ‘C’ in cmH_2_O/l/s

*Y* task completed, *N* task not completed

### Data analysis

**Fig. 2:**
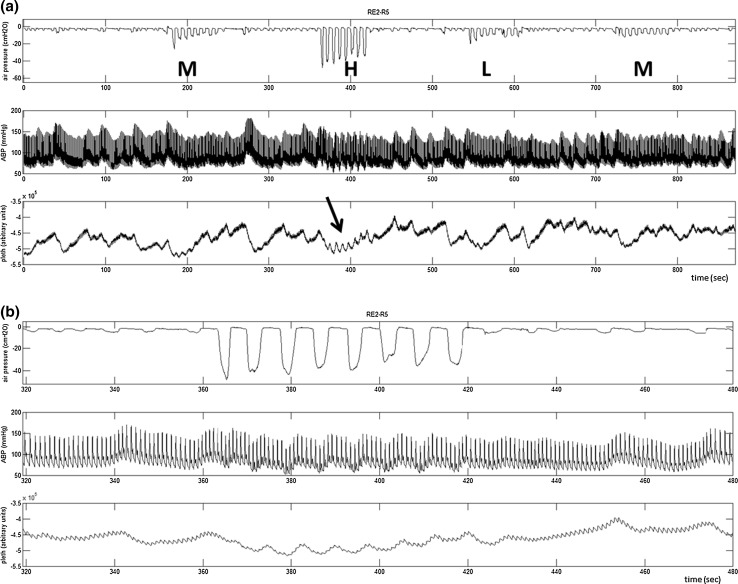
Airflow (*top*), Arterial blood pressure (*middle*) and pleth (*bottom*). **a** Signals for the whole four tasks. **b** Zoom into the signal segments denoted by the *arrow* in (**a**)

An example of the acquired signals is shown in Fig. 2a from one of the task sets for a linear resistor setting of 20 cmH_2_O on exhalation only. The sequential medium, high, low and medium airflow efforts are marked on the plot (M, H, L, M). During these periods the subject breathed at 8 brpm and outside these periods the subject was free to relax and breathe freely at his or her natural rate. Figure 2b shows a zoom of the area marked by an arrow in Fig. 1 where large amplitude modulations can be observed in the pleth signal. It is clear in Fig. 2b that these modulations are also present in the blood pressure signal. DPOP was calculated from the pleth using the algorithmic method described in [30] but with shorter averaging employed as the signal segments were only of 1 min duration (i.e. here DPOP was only averaged over the 1 min window). PPV was computed using the same formula as DPOP. The corresponding air pressure modulation strength (APM) was calculated as the mean of the breath-by-breath maxima of the air pressure modulations. Plots of DPOP versus PPV, DPOP versus APM and PPV versus APM were generated, together with corresponding difference plots.

It can also be observed from the lower plot of Fig. 2b that the respiratory modulations between 380 and 420 s rise up on one of a number of slower waveforms that can be seen in the signal segment. Note that such longer-period modulations are very typical of raw photoplethysmographic signals and are due to a variety of causes: vasomotion, blood pressure changes, slow/slight positional changes or movements, etc. Normally, all such signal information (including the respiratory modulations) is filtered out in standard pulse oximeter devices.

## Results

**Fig. 3:**
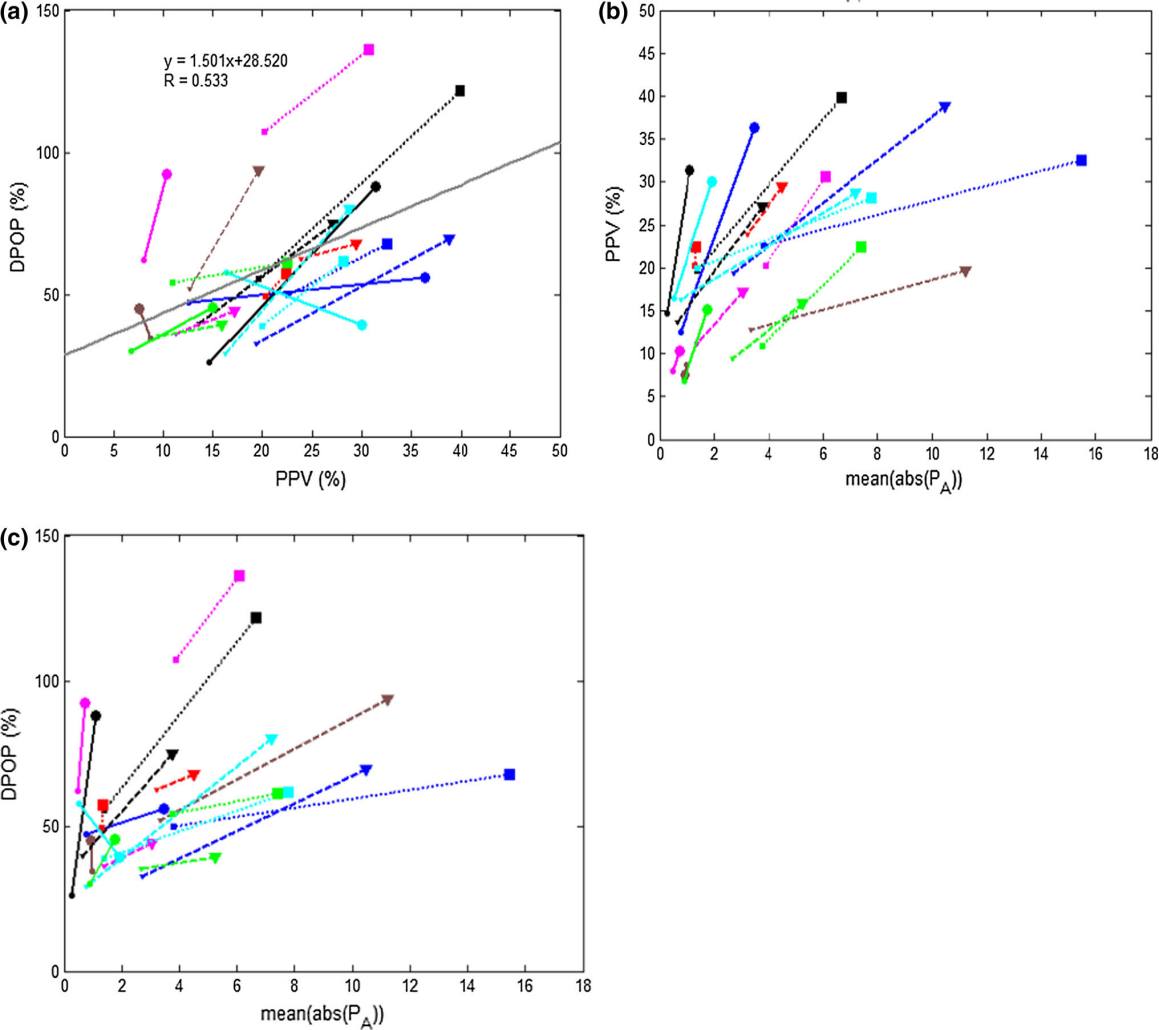
Relationships between airway pressure, DPOP and PPV. **a** Relationship between DPOP and PPV. **b** Relationship between PPV and APM. **c** Relationship between DPOP and APM. Subjects are differentiated by *color*. Linear resistors of 5/20/50 cmH_2_O/l are indicated by *solid*/*dashed*/*dotted lines* with *circles*/*triangles*/*squares* respectively. *High*/*low* breathing efforts are indicated by *large*/*small symbols* at the end of each line. All data were collected with a coached breathing at a respiration rate of 8 brpm

The relationships between APM, PPV and DPOP for the data are shown in Fig. 3. The increasing effort for each task is indicated by linking each of the L–H data pairs by a line where the lower respiratory effort data point is indicated by the smaller symbol and the larger respiratory effort data point by the larger symbol. A least squares fit is shown in Fig. 3a between DPOP and PPV showing the positive correlation with R = 0.533.

**Fig. 4:**
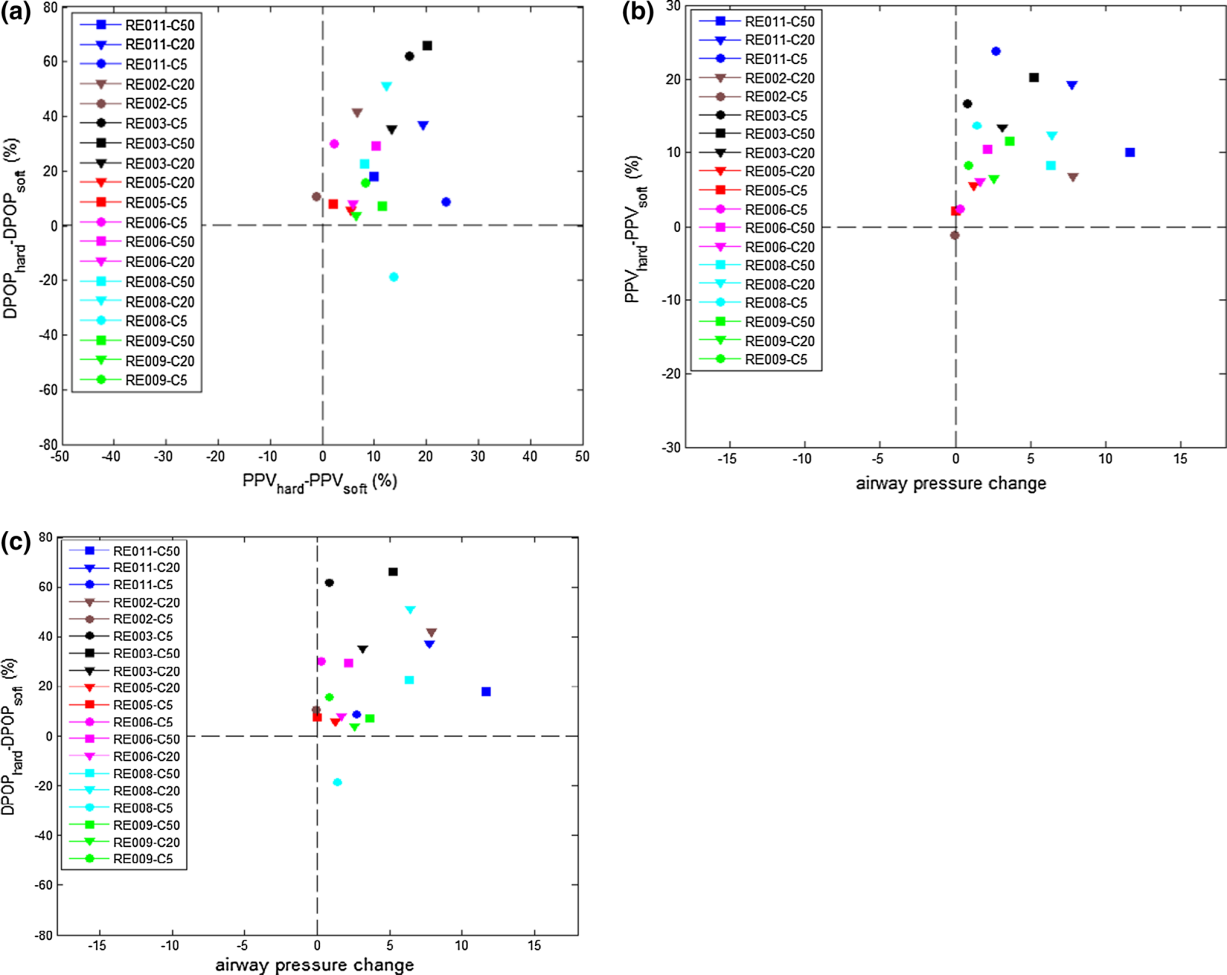
*Difference plots* of respiratory effort induced parameter changes. **a** DPOP change versus PPV change; **b** PPV change versus mean absolute airway pressure change; **c** DPOP change versus mean absolute airway pressure change

Figure 4 contains the difference plots associated with the data in Fig. 3. These comprise the difference in the parameters APM, PPV and DPOP where the parameter values at low subject effort is subtracted from those at high subject effort (small and large symbols in Fig. 3). The plots allow us to see at a glance the general trending direction of the parameters as the subject switches from low effort breathing to high effort breathing. The parameters all appear to increase in magnitude with increasing respiratory effort with the exception of two outliers. The outlier in the lower-right hand quadrant corresponds to a signal acquisition glitch and the one just inside the top-left quadrant corresponds to a relatively low value of change in modulation strength and thus is dominated by signal noise.

**Fig. 5:**
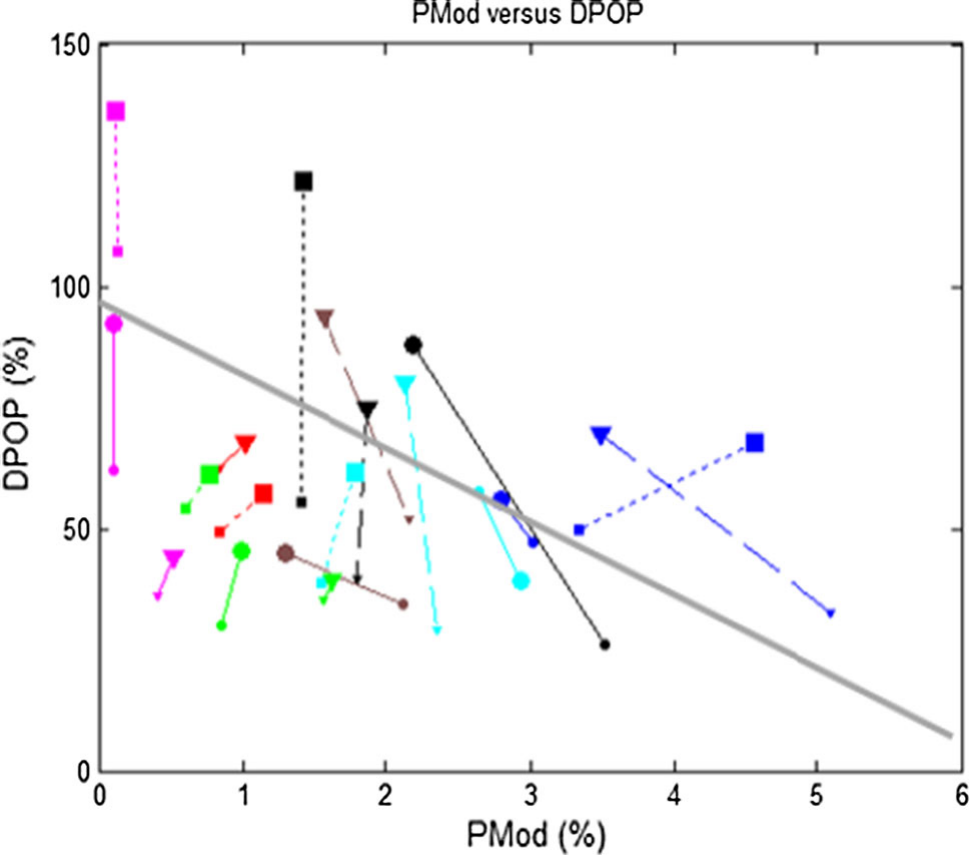
Relationship between DPOP and PMod. Linear resistors of 5/20/50 cmH_2_O/l are indicated by *solid*/*dashed*/*dotted lines* with *circles*/*triangles*/*squares* respectively. *High*/*low* breathing efforts are indicated by *large*/*small symbols* at the end of each line. All data were collected with a coached breathing at a respiration rate of 8 brpm

**Fig. 6:**
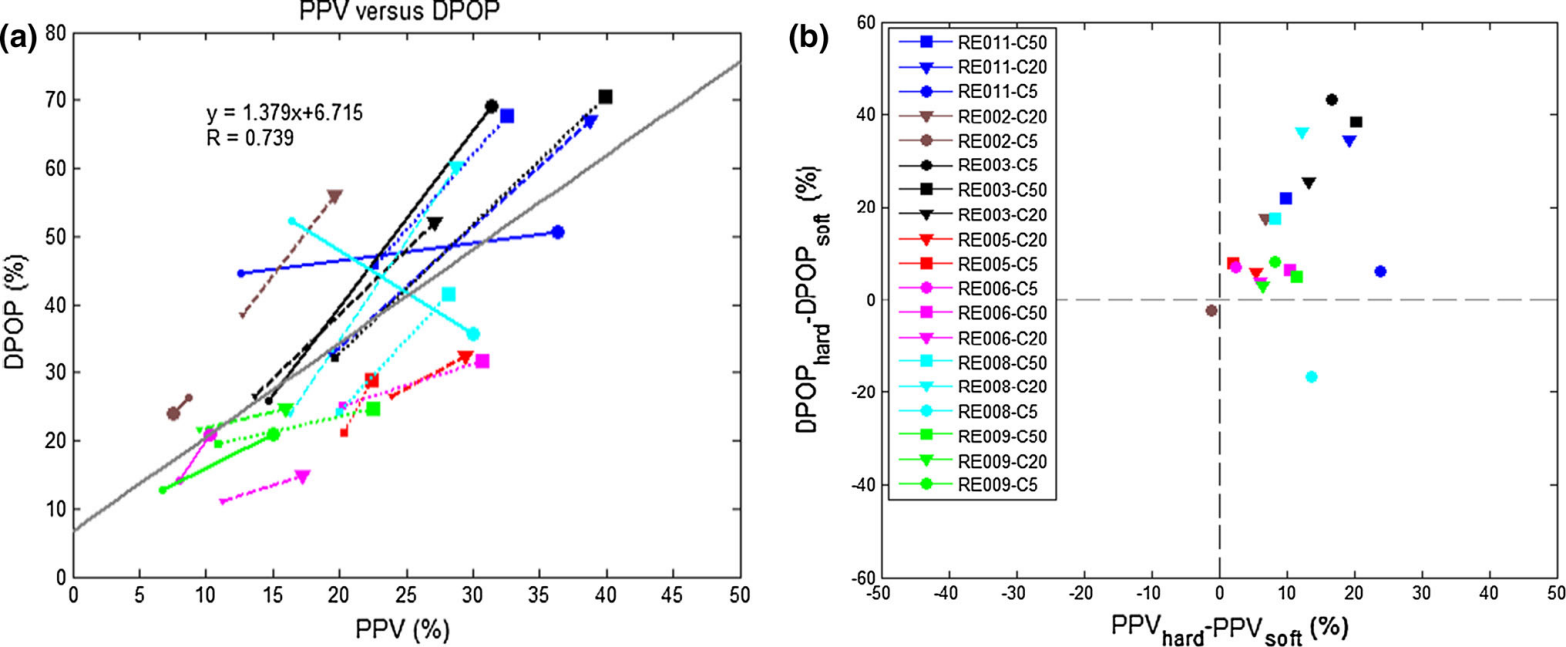
Respiration effort induced change in DPOP and PPV using the modified DPOP algorithm. **a** Relationship between DPOP and PPV, **b** DPOP change versus PPV change

The relationship between DPOP and the percent modulation (PMod) is shown in Fig. 5. PMod, sometimes also called perfusion index, is the ratio of the AC to DC component in the pleth signal. Figure 6 shows the updated version of Figs. 3a and 4a using an enhanced DPOP algorithm with a correction for PMod (described in [31] ). It can be seen that the correlation coefficient between PPV and DPOP has improved from R = 0.533 to 0.739.

## Discussion

Numerous attempts have been made over recent years to assess respiratory effort from the photoplethysmogram. This includes the work by Hartert et al. [16] who used the baseline modulation as a measure of airway obstruction in a group of patients with obstructive airway disease (asthma or COPD). They found that the measure gave good separation when used to identify those patients with abnormal pulsus paradoxus from those with physiologic pulsus (defined either side of a 10 mmHg threshold). Later, Arnold [17] found a relationship between the variation in waveform area and height of the cardiac pulse through the respiratory cycle and changes in mouth pressure in a study of healthy young adults who breathed against progressively increasing levels of mouth pressure. More recently Perel [19] suggested that pleth-based fluid responsiveness measures may be clinically important for the detection of respiratory effort increases due to upper airway obstructions. In fact, the Perel paper prompted the recent editorial by Cannesson and Shelley [32] who asked how the same pulse oximeter waveform could “monitor oxygen saturation, guide fluid-replacement therapy, and spot airway obstruction?” The answer to this lies in two parts. Firstly, oxygen saturation involves a different phenomenological aspect of the photoplethysmogram than the other two where signals from two wavelengths of light are employed and a ratio of normalized cardiac pulse amplitudes is used to derive the parameter. However, fluid responsiveness and the effects of airway obstruction require analysis of the same (respiratory) components in a single signal. The answer to how these can provide both fluid volume status and a respiratory effort measure lies in the dynamics of the patient physiology during the measurement. If breathing at a constant effort then the effect of differing fluid volume levels can be monitored. If however, volume status is held constant—as it is assumed for the subjects in the current study—the effect of changing respiratory efforts may be monitored.

The analysis compared DPOP to PPV. Although the pulsatile pleth and blood pressure waveforms resemble each other, these are not equivalent physiological measures. Nonlinear coupling exists between the pressure pulses and the vessel-wall mechanical activation causing the pulsed light change in the pleth. This is discussed in more detail elsewhere [31]. However, both contain both cardiac and respiratory modulations and both are currently used as fluid responsiveness parameters in practice. Another popular metric for assessing fluid responsiveness through the pleth is the pulse variability index (PVI) parameter. However, as discussed elsewhere by the author, [20] PVI is a proprietary algorithm of the Masimo Corporation (Masimo Corporation, Irvine, CA), and, as such, there is little detail in the literature regarding the signal processing aspects of its implementation. In contrast, the DPOP measure is well known, the author has developed a sophisticated algorithm for its measurement [30, 31] and has access to the proprietary hardware necessary to extract the raw signal required for its computation.

This work has shown that there is an increase in DPOP associated with an increasing effort to breathe. However, this increase is initiated at the subject’s current DPOP value which is subject-specific. How the DPOP value may be used to provide an absolute measure of respiratory effort is something for future work. However, it may have use as a trend indicator, i.e. as an indicator of improving or deteriorating patient respiratory status.

## Conclusions

In the study reported here healthy volunteers were tasked to breathe over a range of respiratory efforts at fixed RR. The subject fluid levels were assumed constant over the short time periods involved and thus would not influence the DPOP value. The results indicate that the increased effort to breathe, in the form of an increase in mouth pressures while breathing against a resistance, manifests itself as an increase in DPOP. Further, a relationship between DPOP and PMod was found. In this regard a version of the DPOP algorithm that corrects for low perfusion was implemented which resulted in an improved relationship between DPOP and PPV.

Although a limited cohort of seven volunteers was used, the results suggest that DPOP may be useful as a respiratory effort parameter, given that the fluid level of the patient is maintained at a constant level over the period of analysis. No apparent difference in results were obtained between those obtained for constriction on inhalation and those for constriction on exhalation. However, this should be tested further with a larger cohort. A suggested next step in the examination of the DPOP parameter as a measure of respiratory effort would be to calculate it continuously during a therapeutic intervention for a respiratory issue: for example, a decrease in DPOP over time may be expected for an asthmatic patient given drug therapy to relieve symptoms. Although normally thought of as a fluid responsiveness parameter, the work suggests that DPOP may have a wider role in patient monitoring. As such, it may more properly be described in the terms first given to it by Cannesson et al. [21], i.e. as a measure of “respiratory variations in pulse oximetry plethysmographic waveform amplitude”. Depending on its mode of use, it may perform the function as a measure of fluid responsiveness, or as suggested by the current work, as a measure of respiratory effort.

## References

[1] Lausted CG, Johnson AT, Scott WH, Johnson MM, Coyne KM, Coursey DC (2006). Maximum static inspiratory and expiratory pressures with different lung volumes. Biomed Eng Online.

[2] Pandit PB, Courtney SE, Pyon KH, Saslow JG, Habib RH (2001). Work of breathing during constant-and variable-flow nasal continuous positive airway pressure in preterm neonates. Pediatrics.

[3] Rapoport DM (2000). Non-invasive detection of respiratory effort-related arousals (RERAs) by a nasal cannula/pressure transducer system. Sleep.

[4] Brock J, Pitson D, Stradling J (1993). Use of pluse transit time as a measure of changes in inspiratory effort. J Ambul Monit.

[5] Pagani J, Villa MP, Calcagnini G, Alterio A, Ambrosio R, Censi F, Ronchetti R (2003). Pulse transit time as a measure of inspiratory effort in children. CHEST J.

[6] Mañanas MA, Alonso JF, Topor ZL, Bruce EN, Houtz P, Caminal P. Frequency parameters from myographic signals for the evaluation of respiratory muscle activity during an increased ventilatory effort. In: Engineering in Medicine and Biology Society, 2003. Proceedings of the 25th annual international conference of the IEEE 2003; 4:3203–3206.

[7] Ertin E, Stohs N, Kumar S, Raij A, al’Absi M, Shah S. AutoSense: unobtrusively wearable sensor suite for inferring the onset, causality, and consequences of stress in the field. In: Proceedings of the 9th ACM conference on embedded networked sensor systems. ACM; 2011. pp. 274–287.

[8] Matecki S, Milesie C, Baleine J, Jacquot A, Cambonie G (2012). Effect of high-flow nasal cannula on nasopharyngeal airway pressure, respiratory muscles loading and respiratory distress symptoms in young infants with severe acute viral bronchiolitis. Eur Respir J.

[9] Sepeku A, Kohi TW (2011). Treatment outcomes of neonatal asphyxia at a national hospital in Dar es Salaam, Tanzania. Afr J Nurs Midwifery.

[10] Nakano H, Hayashi M, Ohshima E, Nishikata N, Shinohara T (2004). Validation of a new system of tracheal sound analysis for the diagnosis of sleep apnea-hypopnea syndrome. Sleep.

[11] Shelley KH (2007). Photoplethysmography: beyond the calculation of arterial oxygen saturation and heart rate. Anesth Analg.

[12] Allen J, Frame JR, Murray A (2002). Microvascular blood flow and skin temperature changes in the fingers following a deep inspiratory gasp. Physiol Meas.

[13] Leonard P, Beattie TF, Addison PS, Watson JN (2003). Standard pulse oximeters can be used to monitor respiratory rate. Emerg Med J.

[14] Addison PS, Watson JN, Mestek ML, Mecca RS (2012). Developing an algorithm for pulse oximetry derived respiratory rate (RRoxi): a healthy volunteer study. J Clin Monit Comput.

[15] Addison PS, Watson JN, Mestek ML, Ochs JP, Uribe AA, Bergese SD. Pulse oximetry-derived respiratory rate in general care floor patients. J Clin Monit Comput. 2014;29(1):113–120. 10.1007/s10877-014-9575-5 PMC4309914 24796734

[16] Hartert TV, Wheeler AP, Sheller JR (1999). Use of pulse oximetry to recognize severity of airflow obstruction in obstructive airway disease: correlation with pulsus paradoxus. CHEST J.

[17] Arnold DH, Spiro DM, Desmond RA, Hagood JS (2005). Estimation of airway obstruction using oximeter plethysmograph waveform data. Respir Res.

[18] Addison PS, Watson JN, Ochs JP, Neitenbach AM, Mestek ML. Flexible pulse oximeter probe design for monitoring respiration parameters: a feasibility demonstration. In: IAMPOV symposium, Yale University, New Haven, CT, 29 June–1 July 2012, Program Syllabus, 2012; pp. 40–41. (keep for now but could replace with A&A paper on resp effort from pleth just submitted).

[19] Perel A (2014). Excessive variations in the plethysmographic waveform during spontaneous ventilation: an important sign of upper airway obstruction. Anesth Analg.

[20] Addison PS (2014). A review of signal processing used in the implementation of the pulse oximetry photoplethysmographic fluid responsiveness parameter. Anesth Analg.

[21] Cannesson M, Besnard C, Durand PG, Bohe J, Jacques D (2005). Relation between respiratory variations in pulse oximetry plethysmographic amplitude and arterial pulse pressure in ventilated patients. Crit Care.

[22] Monnet X, Teboul JL (2013). Assessment of volume responsiveness during mechanical ventilation: recent advances. Crit Care.

[23] Cannesson M, Attof Y, Rosamel P, Desebbe O, Joseph P, Metton O, Bastein O, Lehot J-J (2007). Respiratory variations in pulse oximetry plethysmographic waveform amplitude to predict fluid responsiveness in the operating room. Anesthesiology.

[24] Cannesson M, Desebbe O, Hachemi M, Jacques D, Bastein O, Lehot J-J (2007). Respiratory variations in pulse oximeter waveform amplitude are influenced by venous return in mechanically ventilated patients under general anaesthesia. Eur J Anaesthesiol.

[25] Cannesson M, Delannoy B, Morand A, Rosamel P, Attof Y, Bastein O, Lehot J-J (2008). Does the pleth variability index indicate the respiratory induced variation in the plethysmogram and arterial pressure waveform?. Anesth Analg.

[26] Feissel M, Teboul J-L, Merlani P, Badie J, Faller J-P, Bendjelid K (2007). Plethysmographic dynamic indices predict fluid responsiveness in septic ventilated patients. Intensive Care Med.

[27] Westphal GA, Silva E, Goncalves AR, Filho MC, Figueiredo LFPD (2009). Pulse oximetry wave variation as a non- invasive tool to assess volume status in cardiac surgery. Clinics.

[28] Hoiseth L, Hoff IE, Skare O, Kirkeboen KA, Landsverk SA (2011). Photoplethysmographic and pulse pressure variations during abdominal surgery. Acta Anaesthesiol Scand.

[29] Chandler JR, Cooke E, Petersen C, Karlen W, Froese N, Lim J, Ansermino JM (2012). Pulse oximeter plethysmograph variation and its relationship to the arterial waveform in mechanically ventilated children. J Clin Monit Comput.

[30] Addison PS, Wang R, Uribe AA, Bergese SD (2014). Increasing signal processing sophistication in the calculation of the respiratory modulation of the photoplethysmogram (DPOP). J Clin Monit Comput.

[31] Addison PS, Wang R, McGonigle SJ, Uribe AA, Bergese SD. Calculation of the respiratory modulation of the photoplethysmogram (DPOP) incorporating a correction for low perfusion. Anesthesiol Res Pract. 2014. 10.1155/2014/980149 PMC4142304 25177348

[32] Shelley K, Cannesson M (2014). “Off-label” use of clinical monitors: what happens when new physiologic understanding meets state-of-the-art technology. Anesth Analg.

